# Temperature and food sources influence subadult development and blood-feeding response of *Culicoides obsoletus* (*sensu lato*) under laboratory conditions

**DOI:** 10.1186/s13071-021-04781-8

**Published:** 2021-06-05

**Authors:** Claudia Van den Eynde, Charlotte Sohier, Severine Matthijs, Nick De Regge

**Affiliations:** Sciensano, Enzootic, Vector-Borne and Bee Diseases, Groeselenberg 99, 1180 Brussels, Belgium

**Keywords:** Biting midges, Colony, Vector, Arbovirus, Artificial blood-feeding

## Abstract

**Background:**

*Culicoides obsoletus* (*s.l.*) is the most abundant *Culicoides* species in northern Europe and an important vector of bluetongue virus and Schmallenberg virus. Nevertheless, information on its subadult life stages remains scarce and no laboratory-reared colony exists.

**Methods:**

*C. obsoletus* (*s.l.*) adults were collected in Belgium and transferred to the laboratory in an attempt to establish a laboratory-reared colony. *C. obsoletus* (*s.l.*) were reared from eggs to adults at different temperatures (28 °C, 24 °C, 20/16 °C) and under different food regimes.

**Results:**

The most suitable temperature for rearing seemed to be 24 °C for most developmental parameters, but resulted in a biased 3:1 male/female sex ratio. The latter could be optimized to a 1:1 sex ratio when a 20/16 °C day/night temperature gradient was applied, but rearing at these low temperature conditions resulted in significantly lower egg hatching and pupation rates and a longer subadult development time. Independent of the rearing temperature, adding dung as an additional food source during larval development resulted in a significantly higher adult emergence rate and a decrease in subadult development time. Furthermore, blood-feeding rates of field-collected *C. obsoletus* (*s.l.*) were compared for different blood sources and feeding systems. The overall blood-feeding success was low and only successful with cotton pledgets (2.7% blood-fed midges) and through a membrane system with chicken skin (3.5% blood-fed midges). Higher feeding rates were obtained on cattle blood compared to sheep blood.

**Conclusions:**

These results will help us to determine the necessary conditions to rear a viable laboratory colony of this important vector species, although further optimization is still required.

**Graphical abstract:**

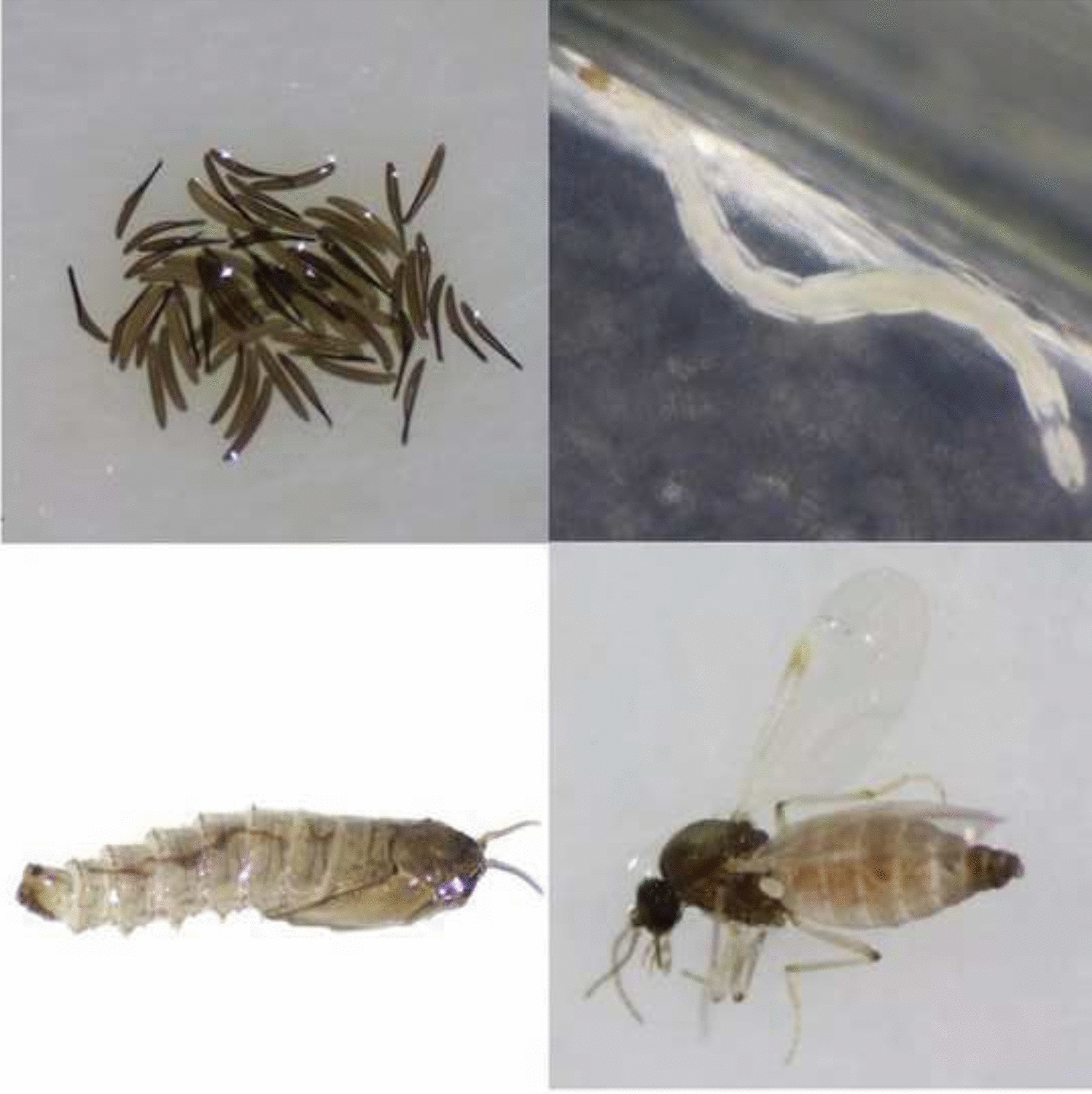

## Background

*Culicoides obsoletus* Meigen and *Culicoides scoticus* Downes and Kettle are the most abundant and widespread *Culicoides* species (Diptera: Ceratopogonidae) in northern Europe [1–3] and the most important vectors of Schmallenberg virus [4] and bluetongue virus [5] in Europe. Both viruses cause serious economic losses to the livestock (cattle, sheep and goats) industry. Since morphological distinction between *C. obsoletus* and *C. scoticus* females is not straightforward, both species are considered together as *C. obsoletus* (*s.l.*). They belong to the subgenus *Avaritia*, together with *Culicoides chiopterus* and *Culicoides dewulfi*.

*C. obsoletus* (*s.l.*) has four larval instars [6] and can overwinter as fourth-instar larvae in diapause [7], indicating that temperature has an influence on larval molting [8] and growth [9]. *C. obsoletus* (*s.l.*) larvae usually inhabit the top few centimetres of moist and highly organic soil substrates (e.g. old, composted manure mixed with soil) usually without freestanding water [6]. *C. obsoletus* (*s.l.*) is an anautogenous species which implies that newly emerged females are not capable of maturing eggs when given sugar solution alone and need a blood meal for this.

Despite the importance of *C. obsoletus* (*s.l.*) as vectors of important veterinary disease agents, no laboratory colonies of *C. obsoletus* (*s.l.*) exist, resulting in very limited information on the life cycle traits of this species, particularly of subadult life stages. Two previous studies described the development from egg to adult on agar for *C. obsoletus*, and reported low survival [10] and biased development towards the production of males [11]. The obstacles to establishing a colony of *C. obsoletus* (*s.l.*) and related species include their reluctance to mate under laboratory conditions [12, 13], high mortality of field-caught midges [14], limited knowledge on their larval nutrition and larval substrates [12] and a reluctance of field-collected females to blood feed under laboratory conditions through artificial blood-feeding systems [12, 15].

The majority of studies of *Culicoides*-arbovirus interactions have been conducted using laboratory lines of *Culicoides sonorensis* infected with either bluetongue virus or the closely related African horse sickness virus [6, 13, 16]. Because this species is relatively large, possesses a rapid life cycle and mates in confined cages under laboratory conditions, it is more straightforward to work with. However, the degree to which the mechanisms of vector competence in *C. sonorensis* are representative of other *Culicoides* vector species is unknown [13]. Therefore, additional research on blood-feeding under laboratory conditions has been conducted with field-collected *C. obsoletus* (*s.l.*), despite their unpredictable availability, using natural and artificial membranes [14, 17–20] with the aim of improving laboratory blood-feeding in order to perform vector competence studies and to attempt to establish a laboratory colony. Existing blood-feeding techniques for *C. obsoletus* (*s.l.*) employ cotton pledgets and a heating system with different membranes, namely chicken skin, Parafilm, Nescofilm and collagen. Differences in feeding rates have been reported depending on the blood-feeding technique, blood source, days of acclimatization and days of starvation before blood-feeding [17].

The aim of our study was to improve our understanding of subadult development and the requirements to establish a viable laboratory colony of *C. obsoletus* (*s.l.*) by (i) evaluating the effect of different combinations of temperature and larval substrates on subadult development and emergence patterns of eggs laid by field-collected midges; and (ii) comparing different methods for artificial blood-feeding of Belgian *C. obsoletus* (*s.l.*)

## Methods

### Collection of *Culicoides*

**Fig. 1:**
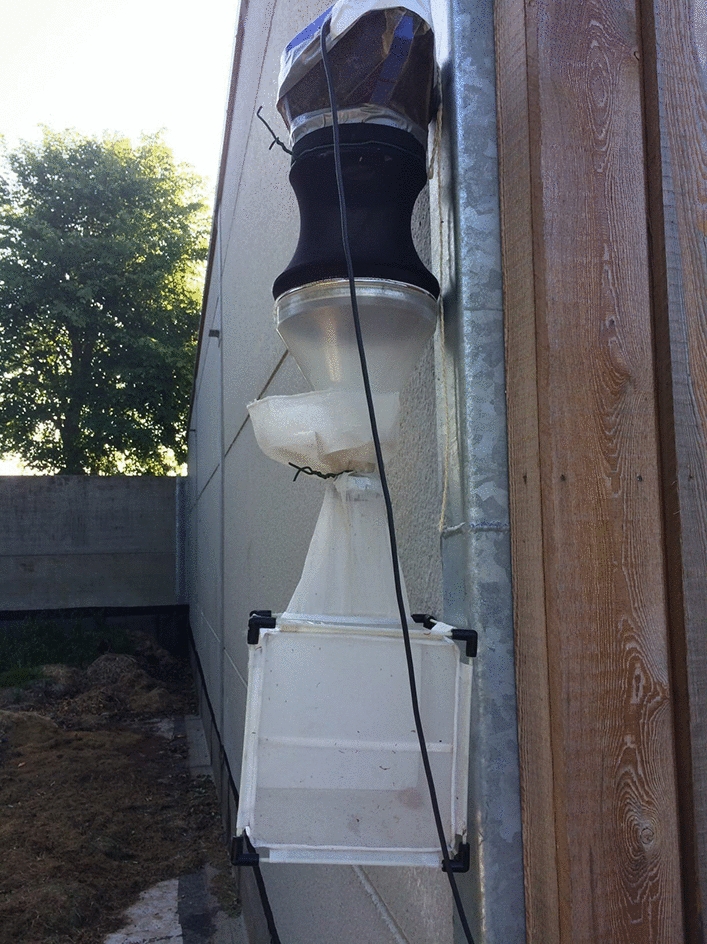
Onderstepoort Veterinary Institute traps modified with a cage (17.5 cm × 17.5 cm × 17.5 cm; BugDorm-41515; MegaView Science, Taichung, Taiwan) to store the live midges

*Culicoides* were collected at cattle and sheep farms in Belgium where abundant *C. obsoletus* (*s.l.*) populations have been found during previous surveillance studies [4, 21]. *Culicoides* were collected with Onderstepoort Veterinary Institute blacklight traps (220 V; 8 W; Onderstepoort, SA), installed outside at 1.5–2 m above ground level, and identified with the *Culicoides* identification key [22]. *Culicoides* were collected on several nights between the end of April and the end of October 2018, 2019 and 2020. The Onderstepoort Veterinary Institute traps were modified with a cage (17.5 cm × 17.5 cm × 17.5 cm; BugDorm-41515; MegaView Science, Taichung, Taiwan) to store the live midges (Fig. 1) and a quarter of a biologically farmed apple was provided to avoid desiccation and as a food source. Traps were operated from 7 p.m. to 8 a.m. and the cages were transferred to the laboratory immediately thereafter to reduce mortality. Once in the laboratory, the piece of apple was removed and a petri dish (Corning; diameter × height 100 mm × 15 mm) containing a patch of cotton wool (Hartmann Watte; Hartmann, Belgium) soaked in 10% sucrose water [18, 23, 24] with a filter paper (Whatman; 110-mm diameter, no. 1001 110) on top was provided as the food source. A second petri dish with a cotton pad soaked in water with a filter paper on top was provided as the oviposition substrate. The *Culicoides* were kept at 24 °C, 80% humidity and 16–8 h light–dark cycle [25].

### Subadult rearing procedures

The development of subadult life stages was studied at three temperature conditions, namely 28 °C, 24 °C and a 20/16 °C day/night temperature gradient, i.e. 16 °C during the 8-h dark (night) period and a temperature increase/decrease rate of 0.27 °C/min at the beginning and end of the 8-h period. A temperature of 28 °C was selected since it has been shown to result in shorter development times and a higher number of offspring of field-caught *Culicoides* (*Avaritia*) *imicola* Kieffer females [12]. To better mimic the temperature conditions of northern Europe, 24 °C and a day/night gradient of 20/16 °C were also tested.

The standard substrate for the subadult rearing consisted of a plastic petri dish filled with 10 ml of 1% liquid broth agar medium [LB Agar, powder (Lennox L agar); Thermo Fisher Scientific, Belgium]. Standard additional food sources for the larvae were provided three times a week and consisted of about 2 mg of the small free-living nematode *Panagrellus redivivus* (acquired from the New Brussels Aquarium, Belgium), maintained on oatmeal (Quaker, Chicago, IL), and 1 ml of the algae *Chlamydomonas reinhardtii* (colony provided by the Department of Plant Biotechnology and Bioinformatics, Ghent University, Belgium), grown in Tris–acetate-phosphate medium in 300-ml flask cultures [26]. *P. redivivus* were reared in plastic boxes on a layer (1 cm) of wet oatmeal. *P. redivivus* were removed directly from the sides of the container, a method adapted from Mullens and Velten [27]. At the 24 °C and 20/16 °C rearing condition (RC), we also studied the effect of adding patches of horse dung to the standard substrate. Horse dung was first maintained at − 20 °C for 48 h to ensure that all arthropods in the material were killed. A clump of ~ 3 g was incorporated into the solidifying agar medium.

Finally, five RCs were compared for subadult development: standard substrate (1% liquid broth agar with nematodes and algae) at 28 °C (RC1); standard substrate at 24 °C (RC2); standard substrate with additional horse dung patches at 24 °C (RC3); standard substrate at a 20/16 °C day/night temperature gradient (RC4); and standard substrate with additional horse dung patches at a 20/16 °C day/night temperature gradient (RC5).

**Fig. 2:**
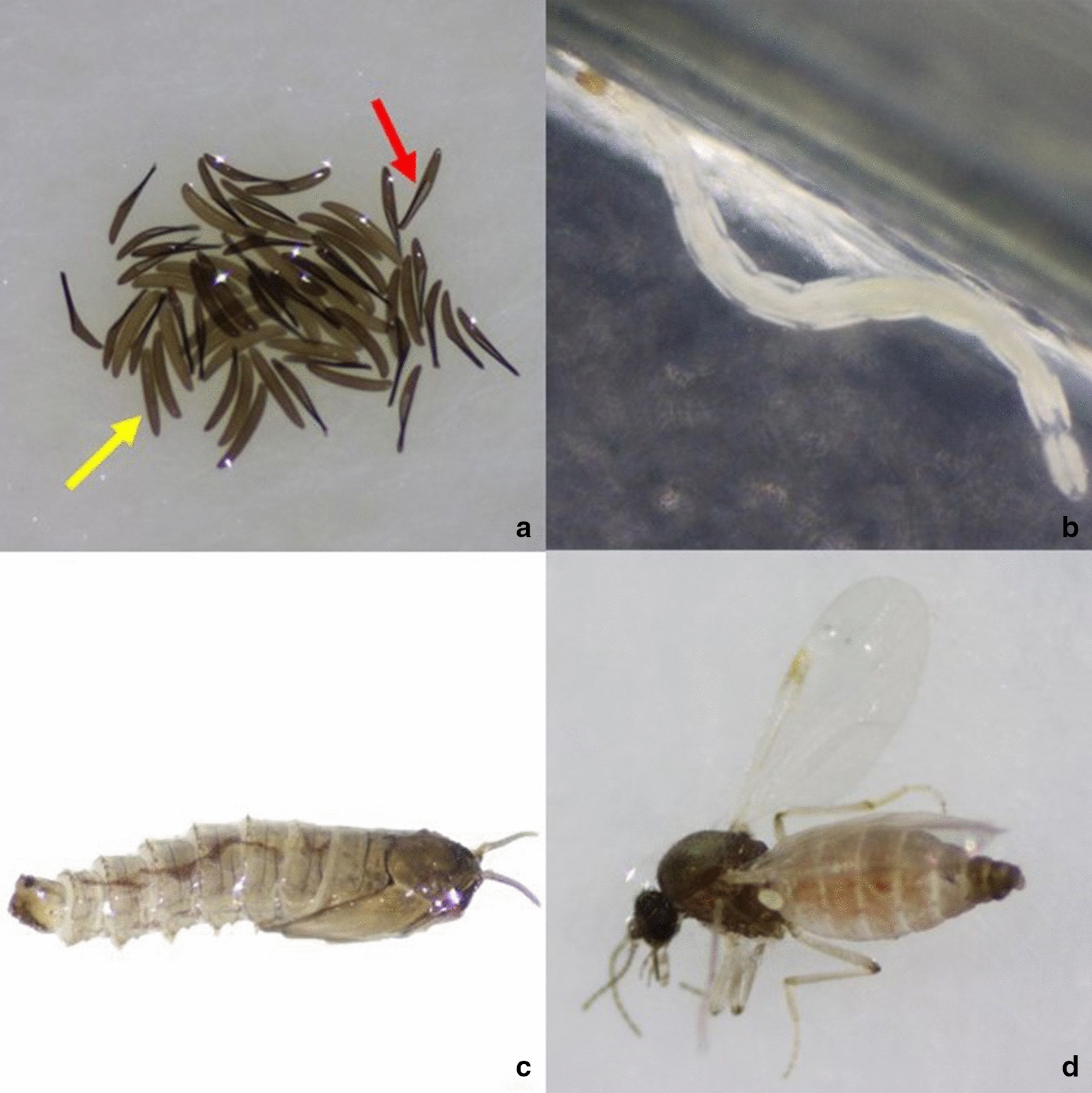
Different developmental stages of *Culicoides obsoletus* (*s.l.*): hatched (*red arrow*) and unhatched (*yellow arrow*) eggs (**a**), fourth-instar larvae (**b**), pupa (**c**) and adult female (**d**)

Eggs were laid by *Culicoides* females that had already blood-fed when collected in the field during the active vector seasons of 2018, 2019 and 2020. After oviposition, the filter paper with eggs was transferred to petri dishes containing the larval substrates described above. If more than 50 eggs were obtained at a specific time point, they were divided between the different RCs. At least 20 repetitions were conducted for each condition. Eggs from 2018 and 2019 were used to study RCs 1–3, while eggs from 2020 were used to study RCs 4 and 5. Since egg batches were mostly limited and the egg hatching rate was low under several conditions, calculating means over the different repeats was not meaningful, and all results per RC were aggregated. *Culicoides* development was followed for 2 months from oviposition onwards and all rearing was performed in an environmental chamber (Panasonic MLR-352H Series; Thermo Fisher Scientific, Belgium) at a 16–8 h light–dark regime and 80% humidity. The day of oviposition of the eggs was designated as day 1. The eggs were counted, placed under a specific RC and observed daily under a stereomicroscope (Zeiss Stemi 508; Zeiss, Belgium) to follow emergence from the eggs and further subadult development. After completion of the fourth larval instar life stage*, C. obsoletus* (*s.l.*) larvae become pale brown pupae (Fig. 2). Pupae were carefully removed from the agar and placed on a moist filter paper on top of a wet cotton wool pad in a container covered with a net, in which they could hatch into adults. The following developmental parameters were recorded: egg hatching rate, larval survival rate (i.e. survival of first-instar larvae, estimated based on the number of hatched eggs, to fourth-instar larvae), pupation rate (i.e. development from fourth-instar larvae to pupae), pupal stage duration, adult emergence rate (i.e. development from pupae to adults) and sex ratio of the emerging adults. Emerged adults were immediately identified and sexed under the stereomicroscope after immobilization with CO_2_.

### Blood collection

Defibrinated blood of sheep and cattle was used for blood-feeding. Defibrination was done following the method of Van der Saag [28]. Fresh blood was collected from cattle and sheep in the slaughterhouse in a flask (2 L) containing 3 layers of glass beads (5-mm diameter, borosilicate; Sigma-Aldrich, Belgium). Immediately upon collection, a gradual swirling motion of the beads was begun by hand at room temperature and continued until defibrination of the blood was complete (5 min). This was indicated by the formation of white foam (fibrin) on top of the blood. Once completed, the defibrinated blood was decanted into sterile screw-top containers (50 ml) and stored at 4 °C.

### *Culicoides* blood-feeding

**Fig. 3:**
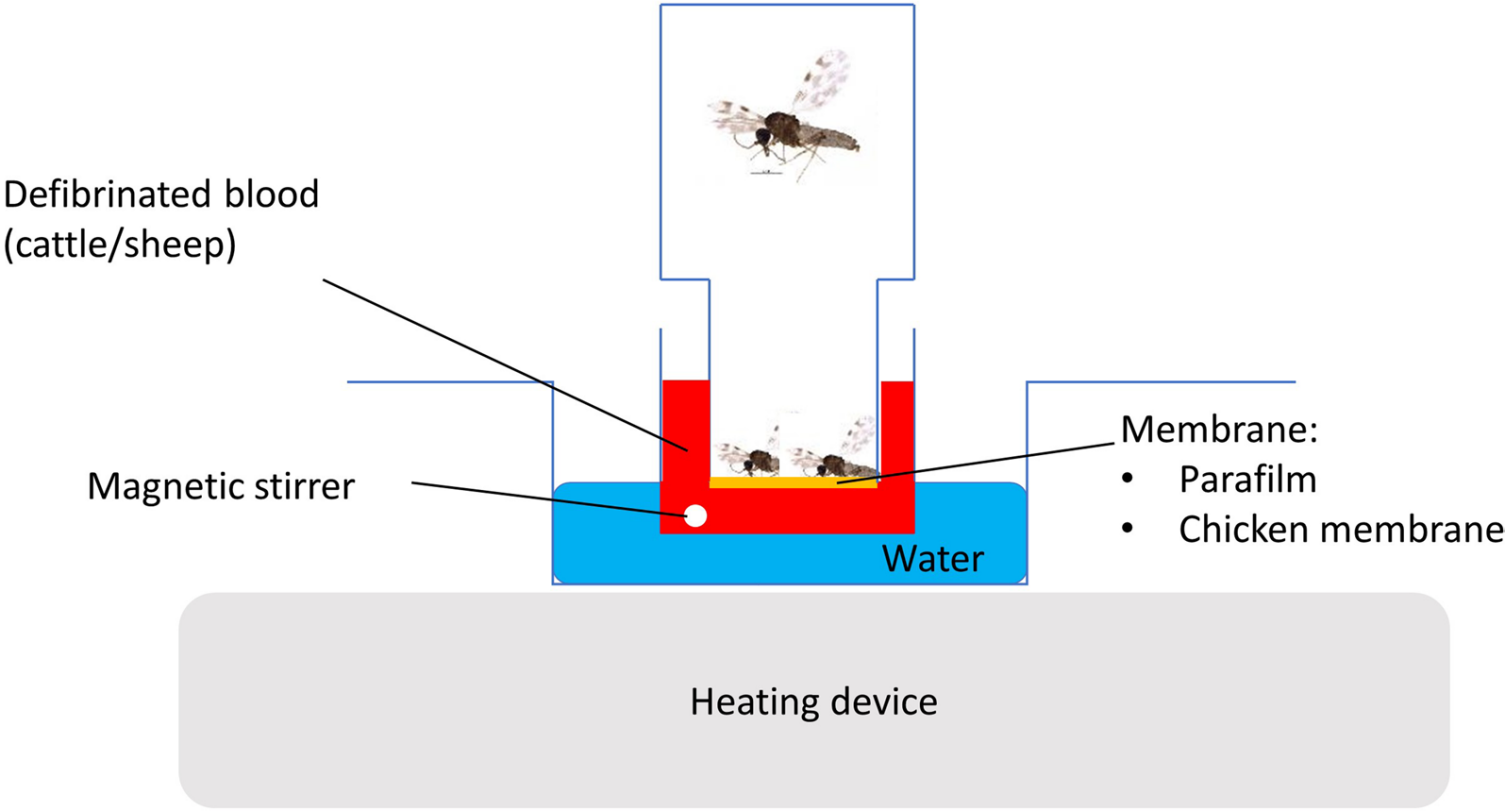
Blood-feeding system as described by Venter et al. [29] with a feeding chamber and a mounted membrane (Parafilm or chicken membrane), container with blood and magnetic stirrer and a water bath on a magnetic heater

In a separate experiment, *Culicoides* were collected for blood-feeding studies and housed in the laboratory as described above. Three days post-collection, the midges were starved for 1 day. At day 5, before placing them into a feeding chamber (200 cm^3^), the live midges were briefly immobilized with CO_2_ to allow species identification and verification of the absence of a previous blood meal (i.e. engorged). Blood of different origins (sheep or cattle blood) and several blood-feeding systems (cotton pledgets soaked in blood [14, 15] and membrane feeding systems) were evaluated. Membrane feeding systems included a stretched Parafilm M membrane (Bermis, USA) or chicken membrane (euthanized 1-day-old chickens were provided by Avian virology and immunology, Sciensano, following national and European regulations, procedure agreement 101105-02, from which the skin was subsequently removed—stretched—and most of the feathers removed) [19, 29, 30] mounted on the blood-feeding system with the feathered side on the inside of the feeding chamber as described by Venter et al. [29] (Fig. 3) or a collagen membrane (Hemotek Feeding Membrane; Hemotek, Blackburn, UK) mounted on the Hemotek system (Hemotek). In the blood-feeding system described by Venter, the blood meal was kept at 37 °C and agitated using a magnetic stirrer (Fig. 3). The feeding chamber consisted of a 40-mm-diameter 50-mm-high plastic bottle. The bottom of the bottle was replaced by the membrane (Parafilm or chicken membrane). The top of the bottle was cut out and replaced by gauze netting [36]. The blood source was placed on the bottom.

The blood meal mounted on the Hemotek system was also kept at 37 °C during feeding. The feeding chamber consisted of a 40-mm-diameter 50-mm-high plastic bottle. The bottom of the bottle was replaced by gauze netting. The top of the bottle was cut out and replaced by gauze netting. The blood source was placed on top of the gauze netting. Depending on the availability of field-collected *Culicoides*, batches of 10–150 *C. obsoletus* (*s.l.*) were fed for 2 h on the systems described above. The number of blood-fed midges was determined after immobilization with CO_2_.

### Statistical analyses

A χ^2^-test and Fisher’s exact test were used to determine whether certain developmental parameters differed significantly between the five RCs. One-way ANOVA was used to determine whether significant differences in the average development time of midges occurred between the different RCs, and an unpaired Student’s *t*-test was used for two-by-two comparisons. Statistical analyses were done using GraphPad Prism 8.3.0. *P*-values < 0.05 were considered to be significant.

## Results

### Development of subadult life stages

Oviposition took place 2–14 days after gravid females were collected in the field. A total of 7510 eggs (Fig. 2) were studied under the five RCs described above. Independent of the rearing temperature, large variations in the hatching rates were observed between individual egg batches (0–100%). Overall, significantly fewer eggs hatched at the lowest temperature condition, namely 14% at the 20/16 °C gradient (RC4) compared to 60% and 87%, respectively at 28 °C (RC1) and 24 °C (RC2) (χ^2^-test RC1-RC2-RC4, χ^2^ = 1315, *df* = 2, *P* < 0.0001; Fisher’s exact test RC1-RC2, RC1-RC4, RC2-RC4) (*P* < 0.0001 in all instances) (Table 1). The addition of dung patches showed no beneficial effect on the egg hatching rates (comparison between RC2 and RC3 and between RC4 and RC5).

**Table 1:** Developmental parameters of *Culicoides obsoletus* (*s.l.*) subadult life stages under five rearing conditions (*RC*s)

	RC1	RC2	RC3	RC4	RC5	Total
Temperature (°C)	28	24	24	20/16 Day/night	20/16 Day/night	
Nematodes/algae	X	X	X	X	X	
Dung patches	–	–	X	–	X	
No. eggs	1905	1748	1852	903	1102	7510
Egg hatching rate (%)	60	87	40	14	8	48
No. hatched eggs	1140	1515	738	129	89	3611
Larval survival rate^a^ (%)	13	15	14	7	16	14
No. larvae	147	224	104	9	14	498
Pupation rate (%)	74	98	91	55.5	86	88.5
No. pupae	109	220	95	5	12	441
Adult emergence rate (%)	39	50	73	40	83	52.5
No. adults	42	109	69	2	10	232
No. ♂ adults	42	83	53	1	5	184
No. ♀ adults	0	26	16	1	5	48
Sex ratio (♂:♀)	1:0	3:1	3:1	1:1	1:1	
Average overall development time (days)	26	26	21	33	30	

*Culicoides* subadult life stages were reared in an incubator under 16/8 h light/dark conditions and 80% relative humidity on 1% liquid broth agar at the indicated temperature and provided with the additional food sources as indicated in the table

^a^Survival from first-instar to fourth-instar larvae

After egg hatching, the highest mortality during subadult development occurred during the development from first- to fourth-instar larvae in all treatments and ranged between 85 and 93%. The addition of horse dung patches significantly increased the larval survival rate from 7 to 16% in the 20/16 °C RC (Fisher’s exact test: *P* = 0.0452). No increase was observed with the addition of dung patches when rearing was done at 24 °C.

Interestingly, the pupation rate (i.e. number of fourth-instar larvae that pupate) varied significantly between the three temperature treatments (χ^2^-test: χ^2^ = 61.30, *df* = 4, *P* < 0.0001) with a lower pupation rate at the higher temperature condition (74% in RC1; Fisher’s exact test RC1-RC2: *P* < 0.0001) and at the lower 20/16 °C day/night temperature gradient (55.5% in RC4; Fisher’s exact test RC4-RC2: *P* < 0.0001) compared to the 24 °C condition (98% in RC2). While the addition of dung patches caused a significant reduction in pupation rate at 24 °C [98–91%; (Fisher’s exact test RC2-RC3: *P* = 0.0052)], it had no significant effect (Fisher’s exact test RC4-RC5: *P* = 0.1616) under the 20/16 °C day/night condition.

No significant differences were recorded between adult emergence rates under the three temperature conditions (χ^2^-test: χ^2^ = 3.630, *df* = 2, *P* = 0.1628). Interestingly, the addition of dung patches increased the adult emergence rate from 50 to 73% (Fisher’s exact test RC2-RC3: *P* = 0.0017) at 24 °C and from 40 to 83% (Fisher’s exact test RC4-RC5: *P* = 0.1165) under the 20/16 °C condition.

Adults emerged within 1–10 days after pupation. The average overall development time varied significantly between the three temperature conditions [one-way ANOVA RC1-RC2-RC4: *F*_(2,158)_ = 8.001, *R*^2^ = 0.09197, *P* = 0.0005] with the longest mean development time of 33 days recorded at 20/16 °C compared to 26 days at 24 °C and 28 °C (Fig. 2; Table 1). The addition of dung patches led to significantly shorter average development times at both temperatures [unpaired *t*-test RC2-RC3: *t*_(216)_ = 2.131, *P* = 0.0342 and RC4-RC5: *t* = 2.462, *df* = 18, *P* = 0.0242].

### Sex ratio of emerging adults

**Fig. 4:**
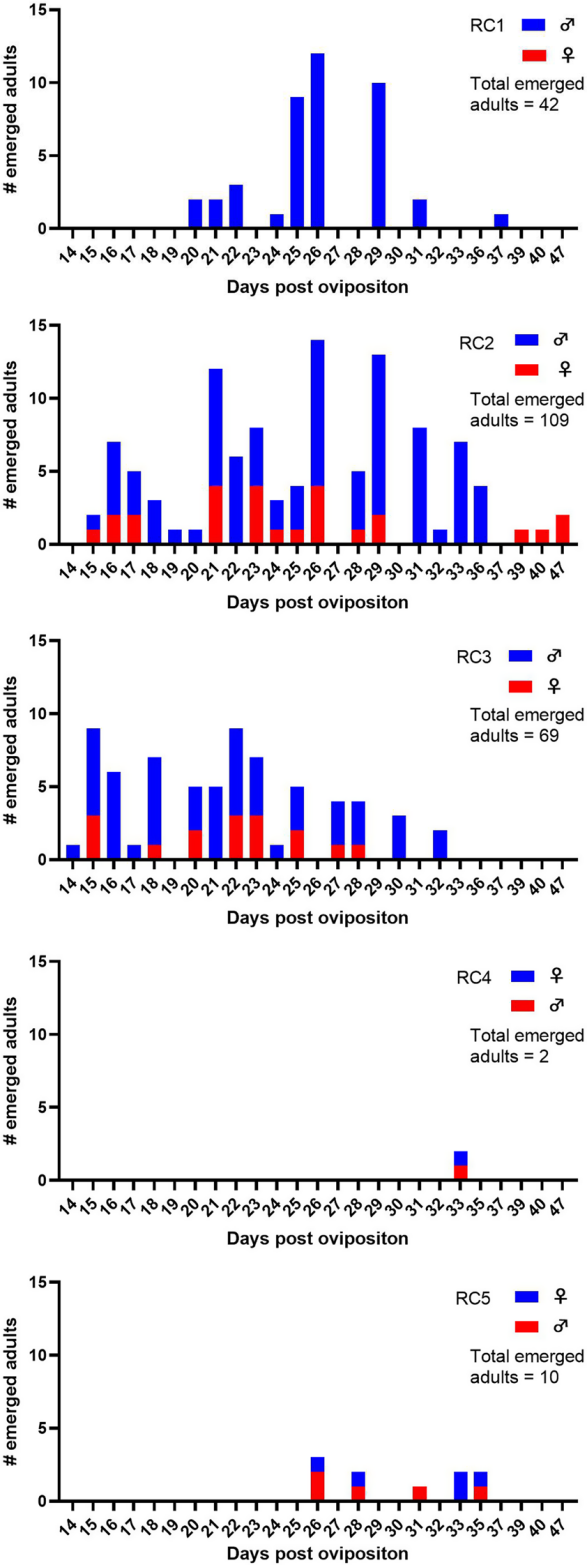
Emergence patterns of male and female *C. obsoletus* (*s.l.*) adults under different rearing conditions [RCs; standard substrate at 28 °C (*RC1*), standard substrate at 24 °C (*RC2*), standard substrate with additional horse dung patches at 24 °C (*RC3*), standard substrate at a 20/16 °C day/night temperature gradient (*RC4*), and standard substrate with additional horse dung patches at a 20/16 °C day/night temperature gradient (*RC5*)]

A bias in sex ratio towards the production of males was observed at 28 °C and 24 °C. Rearing at 28 °C resulted in the complete absence of emerging females. At 24 °C, the sex ratio improved to a 3:1 male/female ratio and the emergence of both sexes occurred over the complete eclosion period (Fig. 4). In contrast, lowering the rearing temperature to a 20/16 °C day/night gradient temperature resulted in an equilibrated 1:1 sex ratio. Food did not seem to impact the sex ratio, since the addition of dung patches did not affect it (Table 1).

### Comparison of blood-feeding methods

A total of 3714 adult C. *obsoletus* (*s.l.*) females were exposed to different blood-feeding systems (cotton pledgets or different membrane types) and different blood sources (sheep or cow blood) to investigate the effect on blood-feeding rates. Overall, engorged midges were obtained in 20 out of 81 (24.7%) blood-feeding attempts, whereby overall feeding rates per feeding system ranged from 1.8 to 4.7% (Table 2). *C. obsoletus* (*s.l.*) could not be induced to feed through a Parafilm membrane mounted on the blood-feeding system described by Venter et al. [29] or through a collagen membrane mounted on the Hemotek system (Table 2). Blood-feeding rates of, respectively, 3.5% and 2.7% were obtained with chicken skin on the blood-feeding system described by Venter et al. [29] and with cotton pledgets. A significantly higher number of *Culicoides* blood-fed on cattle blood than on sheep blood when it was offered with chicken skin (Fisher’s exact test: *P* = 0.005).

**Table 2:** Comparison of effect of different feeding systems and blood source on *C. obsoletus* (*s.l.*) blood-feeding rate

Feeding system	Sheep blood		Cattle blood		Total	
	No. successful/no. total meals	No. engorged/total midges fed (%)	No. successful/no. total meals	No. engorged/total midges fed (%)	No. successful/no. total meals	No. engorged/total midges fed (%)
Pledgets	1/13	20/665 (3.0)	1/6	4/215 (1.8)	2/19	24/880 (2.7)
Collagen	0/5	0/185 (0)	0/5	0/185 (0)	0/10	0/385 (0)
Parafilm	0/5	0/155 (0)	0/4	0/160 (0)	0/9	0/315 (0)
Chicken skin	5/18	25/1063 (2.35)	13/24	50/1071 (4.7)	18/43	75/2134 (3.5)

## Discussion

Currently, limited information is available on several aspects of *C. obsoletus* (*s.l.*) biology, and no viable laboratory colony exists, which hampers research into this significant vector of bluetongue virus and Schmallenberg virus. The encountered obstacles hindering laboratory colonization include a high mortality rate of field-caught midges, limited knowledge on their larval nutrition and larval substrates, and a reluctance of field-collected adults to blood-feed through artificial blood-feeding systems or to mate under captive conditions [15].

In this study, we showed that the life cycle of *C. obsoletus* (*s.l.*) could be completed in the laboratory from eggs (produced by field-collected females) to progeny adults. After bringing field-collected midges to the laboratory, oviposition could be observed for up to 14 days after collection. This delay in oviposition was also recorded for *C. obsoletus* by Hill [31] with a period of up to 18 days, although this is in contrast with the results of Barceló et al. [10] who observed an average time of 4 days for oviposition for field-collected, blood-fed females of *C. obsoletus*.

Independent of the RCs, egg hatching rates of individual egg batches ranged from 0 to 100%. There is no clear explanation for this result, but knowledge from other insect species suggests that low hatch rates could be due to lower fertilization rates when females mate with prior-mated males [32, 33], to the fact that the fertility of the eggs can be reduced after multiple egg-laying cycles, or due to an unknown, laboratory-induced phenomenon [34, 35].

Interestingly, the comparison of RCs for subadult life stages showed that temperature and larval food sources had an important impact on the outcome. The most prominent effect of rearing at the lowest temperature (20/16 °C gradient) was the low egg hatching rate. Since most other developmental parameters were comparable between the different temperature conditions, this resulted in lower numbers of adults at this temperature. A study by Allingham [36] showed a threshold of 16.5 °C for the emergence of *Culicoides brevitarsis* pupae, and this was later confirmed by Bishop et al. [37] who stated that temperatures above 17 °C would allow populations to develop continuously. These findings could possibly help to explain the low egg hatching rate at the lowest temperature conditions in the present study, as the younger stages may be even more sensitive to suboptimal temperatures. A similar effect has been observed in other Dipteran species such as the yellow dung fly, *Scathophaga stercoraria* (Diptera: Scathophagidae). A study on this species revealed reduced egg size and reduced offspring survival even at an intermediate temperature of 24 °C [38].

Other studies reported that higher temperatures resulted in accelerated larval development for different *Culicoides* species, e.g. *C. imicola* [12], *C. sonorensis* [39] and *C. brevitarsis* [36]. This effect of higher temperature was confirmed by our study since the development time from egg to adult *C. obsoletus* (*s.l.*) shortened from 33 days at 20/16 °C to 26 days at 24 °C. However, further increasing the temperature to 28 °C did not result in a shorter development time.

Temperature furthermore affected the sex ratio of emerging adults, with no females found at 28 °C, while 25% of emerging adults were female at 24 °C, and an equal number of males and females emerged at the 20/16 °C temperature gradient. This seems to suggest that sex is not predetermined at fertilization and that environmental conditions, such as temperature, influence the sex ratio of *C. obsoletus* (*s.l.*), although it is not clear at what moment in their development this bias originates. This is in line with another study on *C. obsoletus* [11] that also showed an extreme bias of the sex ratio towards males and suggested temperature as a possible explanation for this. However, the hypothesis that sex is predetermined but males have a better chance, due to an overall shorter development time compared to that of females, to survive and develop to adulthood under suboptimal conditions cannot be excluded. The effect of temperature on sex determination has also been described in other insect species. In *Aedes* mosquitoes, high temperature conditions can cause males to develop into morphological females [40]. In *Sciara* (Diptera: Sciaridae), higher temperatures result in emergence of more females due to changes in genetic imprinting [41].

Other hypotheses, besides a potential influence of temperature, have been put forward to explain biased sex ratios. The effect of insecticides on sex ratio can be ruled out in our RCs as biologically farmed apple (without insecticides) was provided. Various bacterial endosymbionts are known to cause sex ratio distortion in other insect hosts [42]. However, although Lewis et al. [43] screened males and females of various UK species of *Culicoides* for five common sex ratio-distorting endosymbionts (*Cardinium*, *Wolbachia*, *Spiroplasma*, *Arsenophonus* and *Rickettsia*), these endosymbionts were not detected in the *C. obsoletus* group. In our study, no difference in sex ratio was observed when comparing conditions with or without the addition of horse dung, suggesting that environmental bacteria did not play a major role, whereas temperature did lead to significant differences. However, further studies are needed to improve our understanding of parameters influencing sex ratio distortion in *Culicoides*.

Besides temperature, we found that food provided to the larvae also impacted subadult development. The addition of dung as an extra food source led to a higher adult emergence rate from pupae, indicating that this additional food resulted in better developed fourth-instar larvae. Dung probably represents a source of bacteria, which are known to be consumed by *Culicoides* larvae during subadult development [44]. Adding this food source also led to significantly shorter average subadult development at 24 °C and 20/16 °C. The impact of bacteria on larval development should be studied in more detail since it could increase the viability of a future laboratory colony.

These results highlight the difficulty of establishing a laboratory colony of *C. obsoletus* (*s.l.*) from field-collected adults. Future studies should aim not only to produce sufficient adults of both sexes to start a colony of *C. obsoletus* (*s.l.*) but also to improve blood-feeding and mating methods. *C. obsoletus* (*s.l.*) were successfully blood-fed through chicken skin membranes mounted on the blood-feeding system as described by Venter et al. [29] and via cotton pledgets, but with a very low feeding rate. The difficulty of blood-feeding *C. obsoletus* in the laboratory is well known, and low blood-feeding rates have been reported using cotton pledgets or different membranes (Parafilm, Nescofilm and chicken skin) mounted on feeding systems. Our overall feeding success rate was lower than those reported in previous studies, which yielded success rates of 6.5–8% for methods using chicken skin [17, 19], 0.07–6.5% with Parafilm [17, 19, 30] and 9–49% when cotton pledgets were used [28, 30, 45]. Despite previous successful results with Parafilm [17, 19], none of our *Culicoides* could be induced to feed through this membrane. Similar results were found in a recent Italian study that reported feeding rates specific for *C. obsoletus* (*s.l.*) as low as 0.07% with Parafilm [30]. Also, another *Culicoides* species, *C. brevitarsis,* could not be successfully blood-fed using the Hemotek system with Parafilm [28]. We were not able to evaluate Nescofilm since this membrane is no longer commercially available. It could also be interesting to evaluate blood-feeding of *C. obsoletus* (*s.l.*) on embryonated chicken eggs since this led to promising results for Australian *Culicoides* species [28]. Although we obtained different blood-feeding responses with different systems, we did not observe behaviour during the blood meal, nor differences in behaviour when different membranes or systems were used.

Interestingly, a recent study [46] demonstrated that the orientation of the midges during blood-feeding influences their feeding rate. Higher proportions of *C. imicola* fed when the blood reservoir was offered at the bottom of the feeding chamber compared to when blood was offered from above. It will be interesting to evaluate whether their orientation also influences the blood-feeding of *C. obsoletus* (*s.l.*) during future studies.

Since it has been described that *Culicoides* feeding success can be density dependent [47], the most probable explanation for our low blood-feeding rates is the low number of midges that we used per feeding attempt. Whereas most other studies work with large batches of 300–400 midges [47], we could only attempt to blood feed 10–150 midges at a time, due to the low number of *C. obsoletus* that were caught in the field. The temperature of the blood could also influence feeding results. Blood was pre-warmed to 37 °C in accordance with previous studies [17, 19]. A Swiss study, however, obtained better results when the blood was at 25 °C [14] than at 37 °C. This is surprising, as we would expect an inverse response since *C. obsoletus* (*s.l.*) feed on sheep, goats and cattle with a body temperature of around 38.5 °C. Other studies also indicated that the blood-feeding response could be improved if sucrose [28] or ATP [47] were added to the blood. Sucrose and ATP were not used in this study, although these are worth testing in future blood-feeding experiments. However, sucrose has been found to be less than ideal for vector competence experiments [15, 48].

## Conclusion

This study provides insight into the subadult development of C*. obsoletus* (*s.l.*) and clearly shows the impact and importance of temperature and food sources on larval development. Although lower temperatures (20/16 °C gradient) significantly increased the subadult development time and decreased the egg hatching rate, they did result in an appropriate 1:1 male/female sex ratio. Whether this optimal sex ratio was related to development at low temperature in itself or was rather due to the daily fluctuation in temperature under the 20/16 °C condition deserves further study. Since egg hatching, pupation and adult emergence rates were best at 24 °C, we propose testing a 24/20 °C or a 24/16 °C gradient in future studies. It can furthermore be recommended to use rich media for subadult rearing of *C. obsoletus* (*s.l.*) since adding dung as an additional food source significantly increased adult emergence from pupae and shortened the development time. While artificial blood-feeding with chicken skin and cattle blood provided the best results in our experiments, feeding success was low and these methods require further optimization. Issues related to captive mating of *C. obsoletus* will also have to be solved since this species is an obligatory swarmer and its potential swarming and mating cues have not yet been identified. Taken together, these results show that RCs need to be further optimized and several obstacles will need to be tackled before a viable laboratory-reared colony of *C. obsoletus* (*s.l.*) is within reach.

## Data Availability

Not applicable.
